# Longitudinal associations between executive function and positive parenting during early childhood and resilience, self-regulation, and behavioral problems in school-age children

**DOI:** 10.1186/s13034-025-00875-8

**Published:** 2025-03-08

**Authors:** Lalin Rungsattatharm, Priyakorn Tasingha, Pon Trairatvorakul, Weerasak Chonchaiya

**Affiliations:** 1 Division of Growth and Development, Department of Pediatrics, Faculty of Medicine, The Thai Red Cross Society, Chulalongkorn University, and King Chulalongkorn Memorial Hospital, Bangkok, Thailand; 2 Center of Excellence for Maximizing Children’s Developmental Potential, Division of Growth and Development, Department of Pediatrics, Sor Kor Building, 11Th Floor, Faculty of Medicine, Chulalongkorn University, and King Chulalongkorn Memorial Hospital, The Thai Red Cross Society, 1873 Rama IV. Road, Pathumwan, Bangkok, 10330 Thailand

**Keywords:** Behavior, Executive function, Parenting, Resilience, School-age children, Self-regulation

## Abstract

**Background:**

Longitudinal studies of associations between executive function (EF) and parenting behaviors during early childhood and resilience, self-regulation, and behavioral problems in school-age children are scarce. This study aims to evaluate long-term associations between EF and parenting behaviors during preschool and resilience, self-regulatory efficacy, and behavioral problems in 9-year-old children.

**Methods:**

From February 2021-March 2022, 195 participants were enrolled from a larger longitudinal study conducted since age 6 months. Parents reported child EF difficulties and behavioral problems at ages 4, 6, and 9, as well as parenting behaviors at ages 4 and 6. Children reported resilience and self-regulatory efficacy at age 9. The relationships between these variables were analyzed using path analysis.

**Results:**

Among 195 participants (51.3% female) with a median age of 108 months (interquartile range 108–109), EF difficulties at ages 4 and 6 exhibited direct and indirect relationships with lower resilience, reduced self-regulatory efficacy, and behavioral problems at age 9. Positive parenting at ages 4 and 6 were indirectly associated with better resilience, self-regulatory efficacy, and fewer behavioral problems at age 9, mediated by reduced EF difficulties and behavioral problems during preschool.

**Conclusions:**

EF difficulties during preschool were correlated with decreased resilience, self-regulatory efficacy, and behavioral problems in school-age children. Interventions focusing on promoting EF and positive parenting during early childhood may alleviate behavioral problems and potentially enhance resilience and self-regulatory efficacy during school-age.

## Introduction

Executive function (EF) encompasses the brain’s capacity, particularly centered in the prefrontal cortex, to regulate an individual’s thoughts, emotions, and behaviors toward specific goals [1]. Core skills within EF include inhibitory control, working memory, and cognitive flexibility [1], while higher-level EF involves reasoning, problem-solving skills, and planning [1, 2]. EF development begins in infancy, with significant advancements occurring during the preschool period and extend into late adolescence [3]. Notably, EF plays a pivotal role in physical and mental well-being and is essential for lifelong achievements and overall quality of life [1]. During preschool, EF trajectories are marked by rapid growth and variability, which can predict future outcomes in school readiness, self-regulation, social competence, behavioral problems, theory of mind, learning abilities, and academic success [1, 4–7]. Furthermore, within-person changes in EF during early childhood are associated with evolving parenting practices, academic challenges, and the ability of children to adapt to the increasing cognitive and social demands of school [6–8].

EF also influences a wide range of behaviors, including attention control, emotional regulation, social behavior, internalizing, and externalizing behaviors [9, 10]. Children with low self-control often exhibit aggressive and externalizing behavioral problems [11]. Furthermore, EF dysfunctions are linked to various neurodevelopmental and mental health disorders, such as attention-deficit/hyperactivity disorder (ADHD), conduct disorder, delinquency, substance abuse, anxiety, and depression [12–16].

Resilience is a multifaceted concept with varying definitions across different frameworks [17]. From an outcome-oriented perspective, it is generally defined as the ability to adapt and maintain mental or physical well-being despite significant stressors, challenges, or adversities [17]. However, a process-oriented approach conceptualizes resilience as a trajectory characterized by either stable mental health during or after adversity or by temporary disruptions followed by a relatively swift and successful recovery [17]. Alternatively, a trait-oriented perspective defines resilience as a dispositional characteristic or personality trait that enhances an individual’s ability to cope with stress or adversity [17]. Regardless of the framework, resilience serves as a protective resource that shapes individuals’ responses to stress and hardship [17–19]. Associations between EF, particularly working memory and resilience have been observed in adolescents facing poverty and family dysfunction [20]. Furthermore, EF has been associated with dispositional resilience in Latinx children of migrant farm workers [21].

Self-regulatory efficacy refers to the perceived confidence in controlling one’s thoughts, feelings, attention, and actions in various situations [22–24]. This efficacy significantly influences educational development and learning outcomes [25]. Existing literature highlights lower self-regulatory efficacy in academic performance among children with EF dysfunction, particularly those with ADHD, compared to children from the general population [24]. Furthermore, another research found that students with learning disabilities, as well as those with both learning disabilities and comorbid ADHD, reported significantly lower academic self-efficacy beliefs than their neurotypical peers [26].

A previous meta-analysis demonstrated that positive parenting behaviors including warmth, sensitivity, responsiveness, affect, positive regard, support, and physical proximity, as well as cognitive parenting behaviors such as scaffolding, autonomy support, and cognitive stimulation, were associated with higher EF in children aged 0–8 years [27]. Additionally, negative parenting at age 6 was linked to ADHD symptoms in children at age 8, and this association appeared to be partly explained by lower EF at age 6 [28]. Another study also identified EF as a key mediator in the relationship between negative parenting, particularly parental rejection and externalizing behavioral problems in elementary school-aged children [29]. Further evidence from developmental neuroscience and randomized controlled trials (RCTs) underscores the causal impact of parenting behaviors on the development of EF. For example, a recent RCT targeting parental nurturance and sensitivity during infancy demonstrated that such interventions causally influence amygdala-prefrontal cortex (PFC) connectivity and PFC responses to viewing fearful and neutral faces. This study also identified amygdala-PFC connectivity as a potential mediator of the impact of the intervention on child emotion regulation [30]. Similarly, another RCT involving children exposed to neglect or abuse showed that the Attachment and Biobehavioral Catch-up (ABC) intervention, designed to enhance sensitivity and responsiveness during infancy, significantly improved inhibitory control outcomes in middle childhood [31]. These findings highlighted the transformative potential of early parenting interventions on EF development and underscore the role of EF during the preschool years as a potential mediator in the relationship between parenting and child behavioral problems.

Children’s developmental needs and parenting practices evolve significantly from early childhood to school age, along with changes in school success measures and challenging behaviors [7, 8]. Understanding the within-person trajectories of EF starting in preschool is crucial, as these trajectories shape children’s ability to meet developmental milestones and adapt to academic and social demands [7, 8]. Research in a diverse, low-income urban sample revealed that EF develops nonlinearly, with the greatest gains occurring in preschool but significant individual variability still exists [7]. Children with higher EF at preschool entry showed a steeper early growth, while those with slower initial gains grew more rapidly in kindergarten, leading to some convergence in EF trajectories at the end [7]. This convergence highlights the need to investigate how child, family, classroom, and school factors influence variability in EF development [7]. Positive parenting is also a crucial element in fostering the development of resilience and self-regulatory efficacy in children [32].

**Fig. 1:**
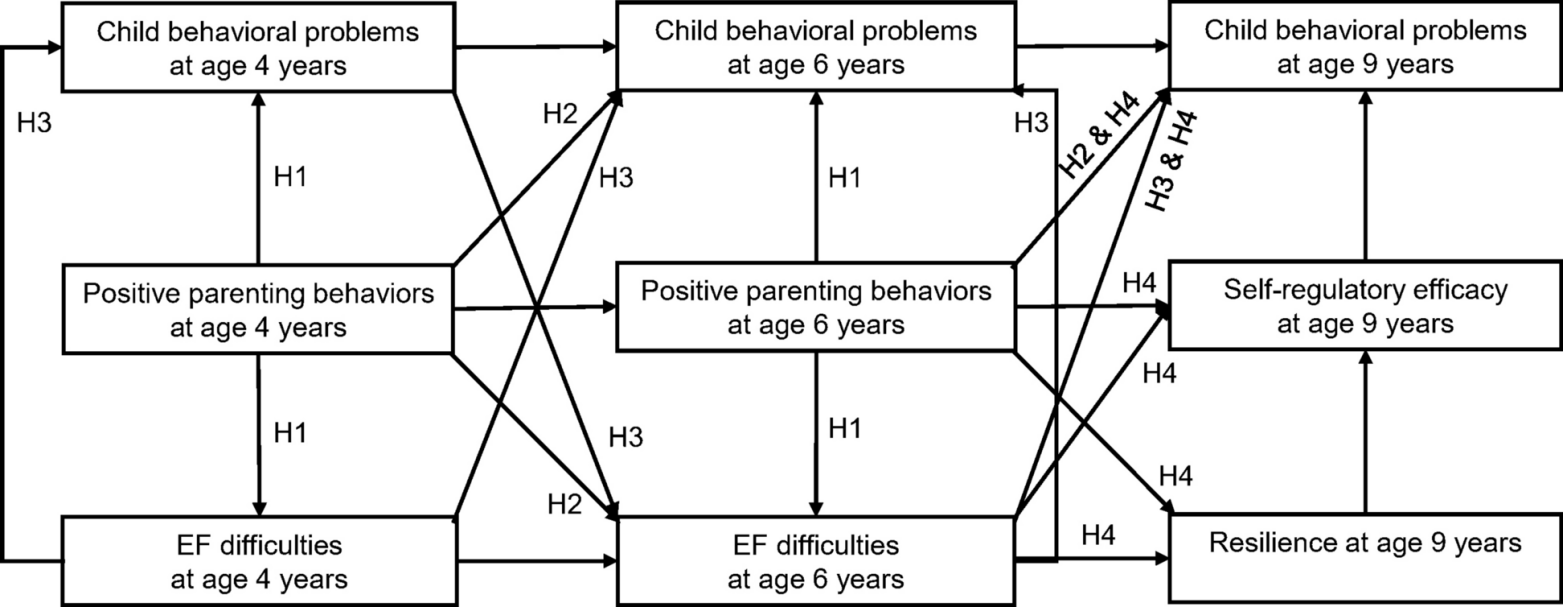
Hypotheses of the study depicting the complex relationships among EF difficulties, positive parenting behaviors, and child behavioral problems at ages 4 and 6, along with their subsequent effects on resilience, self-regulatory efficacy, and behavioral problems at age 9

While cross-sectional studies have linked EF to developmental and behavioral outcomes in older children with impairments, there is a paucity of longitudinal research examining how early childhood EF and parenting behaviors jointly influence resilience, self-regulatory efficacy, and behavioral problems in later childhood. Moreover, few studies have simultaneously modeled these variables to understand their interconnectedness over time. Therefore, this study aims to investigate the long-term associations between EF and parenting behaviors during preschool and resilience, self-regulatory efficacy, and behavioral problems in children at the age of nine. As a result, our hypotheses were as follows (see Fig. 1).

### Hypothesis 1

Children raised by parents who demonstrated more positive parenting styles at ages 4 and 6 would be associated with fewer EF difficulties and behavioral problems compared to those raised by parents who demonstrated fewer positive parenting styles at those same ages.

### Hypothesis 2

Children raised by parents who exhibited more positive parenting styles at earlier ages (4 and 6) would be associated with fewer EF difficulties at age 6 and fewer behavioral problems at later ages (6 and 9) compared to those raised by parents who exhibited fewer positive parenting styles.

### Hypothesis 3

Children with more EF difficulties would exhibit more behavioral problems at the same age. Furthermore, behavioral problems at an earlier age would be associated with EF difficulties at a later age, and vice versa.

### Hypothesis 4

Children with more EF difficulties at earlier ages (age 4 and 6) would demonstrate lower levels of resilience, self-regulatory efficacy, and more behavioral problems at age 9. In contrast, children raised by parents who exhibited more positive parenting styles at ages 4 and 6 would be associated with higher levels of resilience and self-regulatory efficacy, along with fewer behavioral problems compared to those raised by parents who exhibited fewer positive parenting styles.

## Methods

### Participants

Between February 2021 and March 2022, we recruited a total of 195 healthy 9-year-old children who were already enrolled in the primary prospective longitudinal study, “The Impact of Electronic Media Exposure on Young Children Project.” Enrollment in the original study began predominantly at 6 months of age (2012–2014), with additional participants joining at 3 years of age (2015–2016). Detailed descriptions of the original and subsequent studies can be found elsewhere [33–36]. Our participants were from middle to high socioeconomic backgrounds in the Thai context [33–36]. According to our previous study at the age of 3 years, the study enrolled 280 healthy children [34]. Follow-up assessments were conducted when participants were 4 years old (2016–2017) and 6 years old (2018–2019), with retention rates of 97.9% (274 participants) and 92.5% (259 participants), respectively, compared to the cohort of 280 children at 3 years of age [34–36]. At 9 years old, the study aimed to follow all participants from the previous cohort. However, due to constraints including limited researcher availability, concurrent research projects, workload distribution, structural organization for simultaneous research studies, and the COVID-19 pandemic in Thailand at that time, recruitment was concluded in March 2022. Consequently, the study when the participants were 9 years old comprises a sample representing 75.3% of the cohort followed up to age 6. Notably, no significant differences were observed in demographics or primary variables between enrolled and non-enrolled participants. All participants were of Thai ethnicity. The approval for this study was granted by the Institutional Review Board of the Faculty of Medicine, Chulalongkorn University (IRB 007/58). Informed assent was obtained from the participants, while consent was provided by their primary caregivers. This study was conducted in accordance with the principles of the Declaration of Helsinki.

## Measures

### EF difficulties at 4 and 6 years of age

At age 4, parents evaluated EF using the Behavior Rating Inventory of Executive Function-Preschool version (BRIEF-P) for children aged 2 to 5 years [37]. When the children turned 6 years old, the Behavior Rating Inventory of Executive Function (BRIEF) for children aged 5 to 18 years was used [38]. The BRIEF-P comprises 63 items [37], while the BRIEF questionnaire consists of 86 items [38], both designed to assess specific behaviors associated with EF difficulties in children of different age groups. Caregivers rated their children’s behaviors based on the frequency of problematic behavior observed in the previous 6 months, using a scale of never (1), sometimes (2), or often (3).

The 63 items of the BRIEF-P were grouped into five subscales that included inhibit, shift, emotional control, working memory, and plan or organize. Similarly, the 86 items of the BRIEF were categorized into eight subscales, including inhibit, shift, emotional control, initiate, working memory, plan or organize, organization of materials, and monitor. The Global Executive Composite (GEC) served as a total score for EF difficulties, encompassing all EF subscales for each questionnaire and was only used in this study. GEC raw scores were summed and converted into T-scores adjusted for age and sex, with a mean (SD) of 50 (10). A T-score of 65 or higher indicated potential clinical significance [37]. The Cronbach’s alpha of the parent reports ranged from 0.80 to 0.97 for both questionnaires [37, 38], with higher GEC scores indicating greater EF difficulties in this study.

### Parenting styles at 4 and 6 years of age

At 4 and 6 years of age, caregivers of the participants assessed parenting behaviors and styles using the short version of the Parenting Styles and Dimensions Questionnaire (PSDQ short version). The questionnaire comprised 32 items exploring various parenting styles, including authoritative, authoritarian, and permissive approaches. Caregivers indicated how frequently they exhibited behaviors towards the child, with each item rated on a 5-point Likert scale (1 = never, 5 = always). The Cronbach’s alpha of the short version of the PSDQ was 0.86 for authoritative, 0.82 for authoritarian, and 0.64 for permissive parenting styles [39]. In this study, the parenting style was operationalized as a positive parenting score, calculated by subtracting the authoritarian and permissive parenting score from the authoritative parenting score as also used in previous studies [35, 36]. A higher score indicated more positive parenting behaviors.

### Child behavioral problems at 4, 6, and 9 years of age

During the 4-year-old visit, parents completed the Child Behavior Checklist questionnaires for ages 1.5–5 (CBCL 1½–5), comprising 100 items to assess the behaviors of the participants [40]. Each item was scored as not true (0), somewhat or sometimes true (1), or very true or often true (2). CBCL included subscales: emotionally reactive, anxious/depressed, somatic complaints, withdrawn, sleep problems, attention problems, aggressive behavior, and other problems [40]. Subsequently, internalizing, externalizing, and total problem scores were computed, with higher scores indicating more emotional and behavioral problems. A T-score of 63 or higher indicated clinical significance [40]. The Cronbach’s alpha of the CBCL 1½-5 was 0.95 for total problems [40]. Total problems were finally used in the analysis.

At the 6- and 9-year-old visits, parents completed the Strengths and Difficulties Questionnaire (SDQ), comprising 25 items divided into five subscales that included conduct problems, peer problems, hyperactivity/inattention, emotional symptoms, and prosocial behavior [41]. Each item was scored as not true (0), somewhat true (1), or certainly true (2). The first four problem subscales were then calculated as total difficulties scores, with higher scores indicating more behavioral problems. The total difficulties score of less than 16 was defined as having no behavioral problems and was finally used in the analysis. The Cronbach’s alpha of the SDQ was 0.76 for the total difficulties scores [42].

### Resilience

During the 9-year-old visit, children’s resilience was assessed using the Resilience Factors Scale (RFS) questionnaire [43]. The questionnaire consisted of 25 items divided into 6 components, including determination and problem-solving skills, personal support, other kinds of support, positive thinking, assertiveness and balance-of-self, and social skills. Sample items included “I can solve problems in various settings”, “I have people within the family I can trust”, “I have stable family and community”, “I respect myself and others”, “I can express thoughts and feelings without embarrassment”, and “I can negotiate or refuse to do inappropriate things” [43]. Accordingly, this questionnaire measures resilience as a personality trait or dispositional resilience. Each item was rated on a 4-point Likert scale (1 = not true, 4 = true). The total score ranged from 25 to 100, with higher scores indicating greater resilience. The Cronbach’s alpha of this questionnaire was reported to be 0.88 [43]. Permission was obtained from the original authors to adapt the questionnaire for use in this study. Although this questionnaire was originally designed to assess resilience in tenth-to-twelfth graders, it was thoroughly adapted for participants at age 9 years. The content validity was confirmed by three developmental and behavioral pediatricians with extensive experience working with 9-year-olds, yielding an Item Objective Congruence (IOC) index of 0.91. Moreover, Cronbach’s alpha was 0.86 in our current sample, which was comparable to the original research [43].

### Self-regulatory efficacy

During the 9-year-old visit, children’s self-regulatory efficacy was assessed using the Self-Regulatory Efficacy Questionnaire, which is a valid measure in this age group [24]. The questionnaire comprised eight items designed to directly investigate individual’s confidence in their ability to control their attention in various academic contexts, such as the classroom environment, schoolwork, and while taking examinations. For example, participants were asked “How confident are you that you can always focus on school subjects during class?”, “How confident are you that you finish your homework even if it is difficult for you?”, and “How confident are you that you get yourself to prepare for a test?”. Each item was rated on a 5-point Likert scale (1 = I am totally confident I cannot, 5 = I am totally confident I can), resulting in a total score ranging from 8 to 40, with higher scores indicating greater self-regulatory efficacy. The reliability of this questionnaire was reported to be 0.93 [24]. Researchers obtained permission to adapt this questionnaire to the Thai language and implemented it in the current study. Cronbach’s alpha of this questionnaire was 0.80 in our current sample.

### Other variables

Demographic data were collected when participants reached 9 years of age. This included information on the participant’s age, sex, primary caregiver, educational level, type of school, presence of developmental and behavioral problems that required evaluation, management, or counseling by a developmental and behavioral pediatrician, child psychiatrist, or psychologist, and parental marital status. Furthermore, data on parental age and parental education were collected when participants were 3 years old and updated according to the participant’s age at 9 years. These variables were used in the study analysis.

### Statistical analysis

Baseline characteristics were summarized as median and interquartile ranges (IQR) for continuous variables and as percentages for categorical variables. Spearman correlations were used to examine bivariate correlations among demographic characteristics (e.g., age, sex, primary caregiver, and maternal education), EF difficulties at ages 4 and 6, positive parenting behaviors at ages 4 and 6, child behavioral problems at ages 4, 6, and 9 years, as well as resilience and self-regulatory efficacy at 9 years of age. Subsequently, path analysis was performed to assess both direct and indirect pathways of EF difficulties, positive parenting behaviors, as well as child behavioral problems at ages 4 and 6 on resilience, self-regulatory efficacy, and child behavioral problems at 9 years of age while adjusting for confounding factors, such as age, sex, primary caregiver, and maternal education. Path analysis was selected for its ability to simultaneously examine multiple interrelated pathways, offering a clearer understanding of potential mediating effects. The model was specified based on theoretical considerations, with standardized regression weights reported to represent the strength of associations among variables. The fit of the model was assessed using multiple indices, including a non-significant Chi-Square test, a Comparative Fit Index (CFI) ≥ 0.95, a Root Mean Square Error of Approximation (RMSEA) ≤ 0.05, and a Normed Fit Index (NFI) > 0.90, with these thresholds indicating an excellent model fit. All statistical analyses were performed using SPSS and Amos version 28 (IBM Inc., Bangkok, Thailand).

## Results

### Demographic characteristics of study participants

At the 9-year-old visit, this study included 195 healthy participants with a median age of 108 months (IQR 108–109). Of these, 100 (51.3%) were girls, while 95 (48.7%) were boys. Primary caregivers were predominantly mothers (75.6%), followed by fathers (11.4%) and grandmothers (10.4%). Both parents had a median of 16 years of formal education (IQR 16–18), which typically corresponds to at least a bachelor’s degree in the Thai educational context. Most of the participants were enrolled in grade 3 (75.6%), with smaller proportions in grade 4 (22.8%) and grade 2 (1.6%).

Among the 195 participants, 25 (12.8%) exhibited developmental and behavioral problems that required evaluation and management by a developmental and behavioral pediatrician, while three (1.5%) were evaluated and treated by a child psychiatrist. Of the 28 participants with developmental and behavioral problems that required evaluation and management, 18 were diagnosed with ADHD alone, two with specific learning disorder, one with slow learner, one with autism spectrum disorder, one with tic disorder, one with obsessive–compulsive disorder, one with adjustment disorder, and three with dual diagnoses of developmental and behavioral disorders. Additional demographic characteristics of study participants at the age of 9 years are presented in Table 1. The extent of missing data among the 195 participants ranged from 0% to 3.6%, as shown in Tables 1, 2. Given the minimal nature of the missing data, the authors believed that the validity of the longitudinal findings remains robust. The primary findings were not compromised due to the limited extent of missing data observed in the dataset.

**Table 1:** Demographic characteristics of study participants at age 9 years

Characteristics	Total n	n (%)
Child		
Female sex	195	100 (51.3)
Primary caregiver	193	
Mother		146 (75.6)
Father		22 (11.4)
Grandmother		20 (10.4)
Aunt		3 (1.6)
Other		2 (1.0)
Educational level	193	
Grade 2		3 (1.6)
Grade 3		146 (75.6)
Grade 4		44 (22.8)
Type of school	193	
Academic		177 (91.7)
International		8 (4.1)
Other (integrated/ alternative)		8 (4.1)
ADHD diagnosis	193	20 (10.4)
Parent		
Mother’s age (years), median (IQR)	195	43 (40, 45)
Mother’s education (years), median (IQR)	195	16 (16, 18)
Father's age (years, median (IQR)	193	44 (41, 48)
Father’s education (years), median (IQR)	195	16 (16, 18)
Marital status: divorce	191	19 (9.9)

Data presented as median (IQR) for continuous variables and percentage for categorical variables

*IQR* interquartile range

**Table 2:** EF difficulties, positive parenting behaviors, child behavioral problems, resilience, and self-regulatory efficacy at ages 4, 6, and 9 years

Variables	Total n	Age at 4 years median (IQR)	Total n	Age at 6 years median (IQR)	Total n	Age at 9 years median (IQR)
EF difficulties (N < 65)	190	50 (42, 58.3)	188	47 (40.3, 55.8)		
Positive parenting behaviors	190	− 0.4 (− 1.1, 0.5)	188	− 0.03 (− 1.1, 0.8)		
Child behavioral problems on the CBCL (N < 63)	190	50 (42,59)				
Child behavioral problems on the SDQ (N < 16)			188	9 (6, 12)	194	9 (6, 14)
Resilience (25–100)					195	78 (73, 83)
Self− regulatory efficacy (8–40)					195	31 (28, 35)

*IQR* interquartile range

### EF difficulties, positive parenting behaviors, child behavioral problems, resilience, and self-regulatory efficacy at ages 4, 6, and 9 years

Most of the participants demonstrated Global Executive Composite (GEC) scores within the normal range (GEC score < 65) at the ages of 4 and 6 years, as indicated in Table 2. Among the 190 participants with EF data at the 4-year visit, 29 (15.3%) had GEC scores in the range of potential clinical significance, while 15 of 188 (8.0%) had EF difficulties at the age of 6 years. Similarly, most of the participants exhibited behavioral problem scores within the normal range on the Child Behavior Checklist (CBCL) (total problem scores < 63) at 4 years of age and on the Strengths and Difficulties Questionnaire (SDQ) (total difficulties score < 16) at 6 and 9 years of age. However, 35 (18.4%), 28 (14.9%), and 34 (17.5%) participants had behavioral difficulty scores above the cutoff at the ages of 4, 6, and 9 years, respectively. Participants themselves reported relatively high resilience scores and self-regulatory efficacy at the age of 9 years. More details on the median and interquartile range (IQR) of EF difficulties, positive parenting behaviors, child behavioral problems, resilience, and self-regulatory efficacy at ages 4, 6, and 9 years are presented in Table 2.

### Bivariate correlations among key variables

Table 3 presents bivariate correlations between demographic characteristics, EF difficulties at ages 4 and 6, positive parenting behaviors at ages 4 and 6, child behavioral problems at ages 4, 6, and 9, as well as resilience and self-regulatory efficacy at age 9.

**Table 3:** Bivariate correlations among key constructs

Variables	1	2	3	4	5	6	7	8	9	10	11	12
1. Age at 9 years (months)	–											
2. Male sex	0.02	–										
3. Mother’s education (years)	− 0.16*	− 0.10	−									
4. EF difficulties at age 4 years	0.06	0.29**	− 0.27**	−								
5. EF difficulties at age 6 years	0.11	0.19**	− 0.10	0.62**	−							
6. Positive parenting behaviors at age 4 years	− 0.01	− 0.09	0.28**	− 0.51**	− 0.36**	−						
7. Positive parenting behaviors at age 6 years	− 0.04	− 0.19**	0.15*	− 0.44**	− 0.52**	0.59**	−					
8. Child behavioral problems at age 4 years	0.02	0.11	− 0.22**	0.80**	0.57**	− 0.55**	− 0.48**	−				
9. Child behavioral problems at age 6 years	0.03	0.18*	− 0.12	0.53**	0.70**	− 0.39**	− 0.54**	0.58**	−			
10. Child behavioral problems at age 9 years	0.00	0.21**	− 0.15*	0.46**	0.57**	− 0.39**	− 0.37**	0.44**	0.63**	–		
11. Resilience at age 9 years	− 0.05	− 0.09	− 0.01	− 0.14	− 0.17*	0.10	0.03	− 0.13	− 0.18*	− 0.22**	–	–
12. Self-regulatory efficacy at age 9 years	0.02	0.05	0.05	− 0.04	− 0.06	0.04	0.00	− 0.04	− 0.10	− 0.17*	0.63**	–

^*^p < 0.05, **p < 0.01

No significant associations were found between age and other variables. However, boys showed a higher likelihood of EF difficulties at ages 4 and 6, fewer positive parenting behaviors at age 6, and increased behavioral problems at ages 6 and 9. Higher maternal education was related to fewer EF difficulties at age 4, reduced child behavioral problems at ages 4 and 9, and more positive parenting behaviors at ages 4 and 6.

EF difficulties at 6 years of age were associated with lower resilience at age 9. Similarly, child behavioral problems at ages 6 and 9 were associated with reduced resilience, while only behavioral problems at age 9 were associated with lower self-regulatory efficacy at the same age. Additionally, self-regulatory efficacy showed a strong positive association with resilience at age 9. Further bivariate correlations among key variables are presented in Table 3.

### Path analysis

Although high bivariate correlations suggested strong associations according to Table 3, they were accounted for in our path analysis model. Specifically, the model included these variables as distinct constructs with separate pathways, which reduced multicollinearity and ensured that each variable’s unique contribution to the outcomes was assessed.

**Fig. 2:**
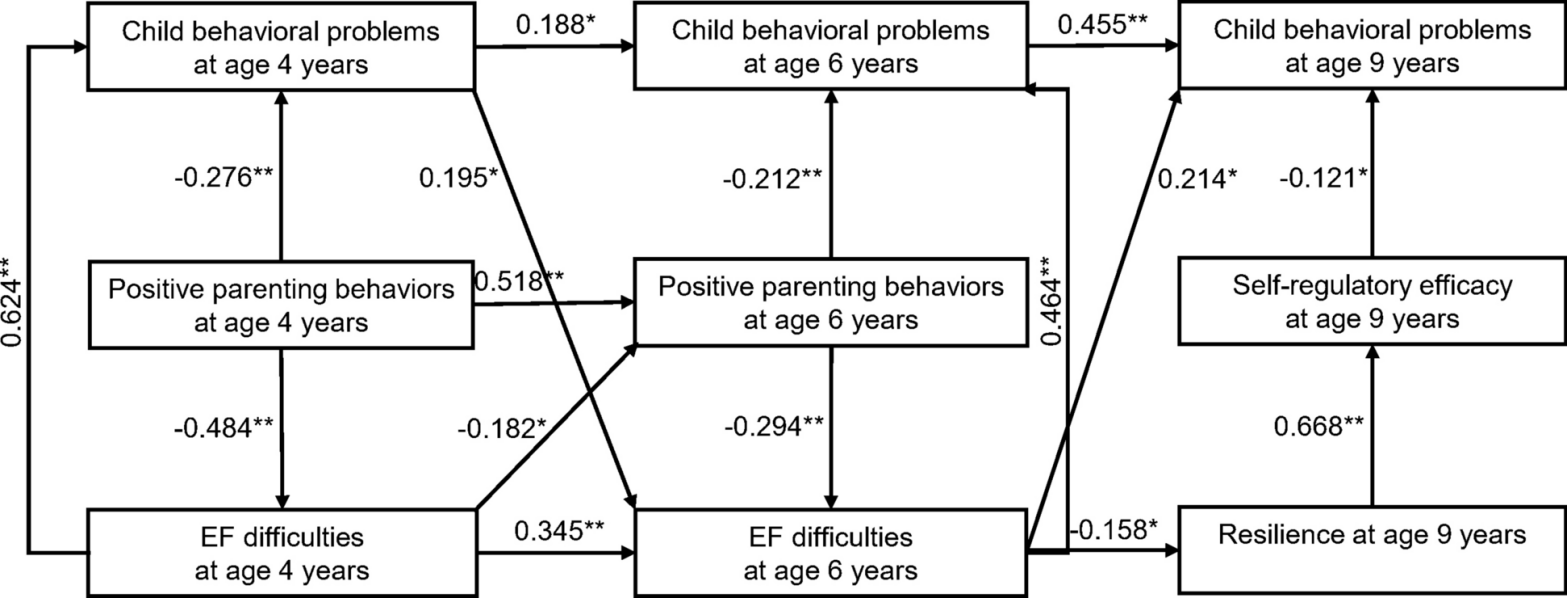
Path analysis model showing the complex direct and indirect relationships of EF difficulties, positive parenting behaviors, child behavioral problems at ages 4 and 6, and subsequent outcomes of resilience, self-regulatory efficacy, and child behavioral problems at 9 years of age adjusted for the child’s age, sex, primary caregiver, and maternal education. Chi-square = 22.417 (d.f. 20), *p* = 0.318, Comparative Fit Index (CFI) = 0.997, Root Mean Square Error of Approximation (RMSEA) = 0.025, Normed Fit Index (NFI) = 0.973. *p < 0.05 and **p < 0.001

Figure 2 presents a path analysis model that shows the complex direct and indirect relationships of EF difficulties, positive parenting behaviors, child behavioral problems at ages 4 and 6, and subsequent outcomes at age 9, including resilience, self-regulatory efficacy, and child behavioral problems. The model was adjusted for age, sex, primary caregiver, and maternal education, and demonstrated an excellent fit: Chi-square (d.f. 20) = 22.417, *p* = 0.318, CFI = 0.997, RMSEA = 0.025, and NFI = 0.973, indicating strong model performance. The analysis revealed several key findings. EF difficulties, positive parenting behaviors, and child behavioral problems at 4 years of age displayed positive direct relationships with the same variables at later ages, as expected.

Consistent with Hypothesis 1, our results showed that positive parenting styles at ages 4 and 6 were directly associated with lower EF difficulties and fewer child behavioral problems compared to those raised by parents who demonstrated fewer positive parenting styles at those same ages.

Regarding Hypothesis 2, instead of direct relationships, we found that children whose parents exhibited more positive parenting styles at age 4 experienced fewer EF difficulties at age 6 compared to those raised by parents who exhibited fewer positive parenting styles. This indirect effect was mediated by reduced EF difficulties at age 4 or fewer positive parenting styles at age 6. Furthermore, children raised by parents who demonstrated more positive parenting styles at ages 4 and 6 had fewer behavioral problems at ages 6 and 9 compared to those raised by parents who exhibited fewer positive parenting styles, respectively, mediated by reduced behavioral problems at ages 4 and 6.

In line with Hypothesis 3, we found that EF difficulties were positively directly associated with behavioral problems at the same ages. Additionally, child behavioral problems at age 4 were directly associated with EF difficulties at age 6. However, EF difficulties at age 4 did not have a direct association with child behavioral problems at age 6; instead, they had an indirect relationship mediated by reduced positive parenting behaviors at age 6. On the other hand, EF difficulties at age 6, were directly associated with behavioral problems at age 9.

According to Hypothesis 4, EF difficulties at age 6 had a direct negative relationship with resilience at age 9. Additionally, EF difficulties at ages 4 and 6 were indirectly associated with self-regulatory efficacy at age 9, mediated by lower resilience. EF difficulties at both ages also had indirect relationships with child behavioral problems at age 9, mediated by reduced resilience and lower self-regulatory efficacy at age 9, or by increased behavioral problems at ages 4 and 6.

Positive parenting behaviors at ages 4 and 6 had indirect relationships with resilience and child behavioral problems at age 9, mediated by lower EF difficulties and fewer behavioral problems, respectively, at earlier ages. On the contrary, reduced positive parenting behaviors at ages 4 and 6 were indirectly associated with behavioral problems at age 9, through pathways involving increased EF difficulties at ages 4 and 6, reduced resilience, and lower self-regulatory efficacy at age 9, or through behavioral problems at ages 4 and 6. Similarly, positive parenting behaviors at ages 4 and 6 were indirectly associated with self-regulatory efficacy at age 9, mediated by lower EF difficulties at earlier ages and higher resilience at age 9.

## Discussion

To our knowledge, this is the first study to longitudinally explore the intricate relationships between executive function and parenting behaviors during early childhood, and their subsequent impacts on resilience, self-regulatory efficacy, and behavioral problems in school-age children. In particular, we investigated these relationships within a single model, taking into account various confounding factors, including age, sex, primary caregiver, and maternal education. Our main findings revealed that EF difficulties at ages 4 and 6 exhibited direct and indirect relationships with decreased resilience, decreased self-regulatory efficacy, and increased behavioral problems at age 9. Concurrently, positive parenting behaviors at ages 4 and 6 were indirectly associated with enhanced resilience, heightened self-regulatory efficacy, and reduced behavioral problems at age 9. These associations were mediated through the alleviation of EF difficulties and fewer behavioral problems during the preschool years. Consistent with our hypotheses, individuals exhibiting decreased EF difficulties and those nurtured by parents who demonstrated positive parenting behaviors since preschool showed elevated levels of resilience, self-regulatory efficacy, and fewer behavioral problems during their school years. Although the existing literature has focused on the correlation between EF difficulties and reduced resilience in individuals exposed to adverse childhood experiences and the underserved population [19–21, 44, 45], our study extends these findings to a cohort primarily consisting of individuals of middle to high socioeconomic backgrounds, with fewer instances of early life adversities. Therefore, our results underscore the critical importance of fostering the development of EF during the preschool period, even among children with a lower risk of adverse childhood experiences, as it can significantly improve resilience in typically developing school-age children.

Our finding on the association between EF difficulties and reduced self-regulatory efficacy in healthy individuals aligns with previous research conducted among individuals with attention and EF problems, as well as learning disabilities [24, 26]. This consistency may be attributed to the evaluation of self-regulatory efficacy in academic tasks and school-related activities in our study, which encompassed various components of EF, including attention and interference control, executive function, and emotion regulation [24]. Furthermore, the moderate to strong correlation observed between self-regulation and resilience in previous studies [21, 45], as well as in our own findings, suggests that individuals with greater resilience demonstrate a greater ability to adapt their behaviors in response to challenges, particularly within academic settings. Therefore, an indirect relationship between EF difficulties during preschool and decreased self-regulatory efficacy in school-age children observed in this study could be explained by a mediation pathway through lower resilience, consistent with findings from another cross-sectional study [21]. Overall, these results underscore the intricate interplay between EF, self-regulatory efficacy, and resilience, highlighting the importance of addressing EF difficulties early in life to promote better resilience and self-regulation in school-age children.

Unsurprisingly, EF difficulties were directly associated with child behavioral problems at the same age [37, 38]. Furthermore, behavioral problems in children at 4 years of age were directly related to EF difficulties at 6 years of age, and this latter variable was also associated with child behavioral problems at age 9, suggesting a bidirectional relationship between child behavioral problems and EF difficulties across development. As expected, positive parenting behaviors during preschool had direct negative relationships with EF difficulties and behavioral problems at the same age, consistent with previous studies [8, 27, 36]. However, these relationships were stronger for positive parenting behaviors and decreased EF difficulties compared to the association between positive parenting styles and fewer behavioral problems. These patterns were also more pronounced at age 4 than at age 6. Similarly, the relationship between EF difficulties and child behavioral problems at the same age was more robust at age 4 than at age 6. Previous research also demonstrated a significantly stronger association between parenting behaviors and EF, as well as behavioral problems in younger children than in older ones [8, 27, 36]. These discrepancies during early preschool and the transition from preschool to school age may be due to developmental changes in both parenting behaviors and the ability of children to regulate behavior. Parenting strategies often evolve as children grow and adapt to the developmental needs of the child. For instance, younger children (around age 3 to 4) commonly exhibit typical oppositional behaviors, which can prompt parents to employ more structured and directive strategies to manage such behaviors. These strategies may have a more immediate impact on both EF and behavioral outcomes during early childhood. By the time children reach early school age, factors such as increased cognitive maturity, school experiences, peer influence, and improved self-regulation often reduce the direct influence of parenting behaviors on EF and behavioral outcomes. As children gain greater self-control and cognitive flexibility, they become more capable of understanding and internalizing parental expectations, which may contribute to the weaker associations observed in the older age group. This developmental perspective underscores the importance of early positive parenting interventions aimed at fostering EF and behavioral regulation during the preschool years, when parenting behaviors may exert a stronger influence on developmental outcomes.

In addition to the relationship between positive parenting behaviors and decreased EF difficulties observed in this study, EF difficulties at age 4 were found to have a direct negative relationship with positive parenting behaviors at age 6. This finding highlights the potential bidirectional relationship between EF and parenting, as reported in previous literature [8]. Furthermore, similar associations between EF difficulties and disruptions in parenting behaviors have been observed in children with excessive screen media use, further supporting this bidirectional framework. Studies have shown that increased and inappropriate exposure to screen media in early childhood is associated with a decrease in positive parenting behaviors over time, and vice versa [34–36]. For example, longitudinal data have indicated that greater screen time in early childhood can disrupt parent–child interactions, leading to less parental responsiveness and warmth [34, 46]. This disruption may, in turn, contribute to a reduction in positive parenting practices as children grow. Additionally, excessive and inappropriate use of screen media is associated with poor self-control and EF difficulties, further compounding these negative effects [47–49]. Therefore, the supporting evidence from previous research and our study underscores the importance of a positive parenting style as a crucial factor in promoting greater resilience, improved self-regulatory efficacy, and reduced problematic behaviors, including optimal use of screen media, in school-age children. These effects were mediated by the attenuation of EF difficulties and fewer behavioral problems during the preschool years. This finding is consistent with previous literature that has consistently highlighted the importance of secure attachment relationships, synchronization within parent–child relationships, the provision of stable environments, and effective caregiving and parenting to foster resilience, improve self-regulation, and promote positive behaviors in children [18, 19, 27, 32, 45].

Moreover, the quality of the early child rearing environment has been identified as a positive determinant of the development of EF and well-regulated behaviors later in life [27, 36, 50]. Positive parenting behaviors, including warmth, sensitivity, responsiveness, affect, positive regard, support, and physical proximity, as well as cognitive parenting behaviors, such as scaffolding, autonomy support, and cognitive stimulation, were associated with higher EF in children aged 0–8 years. In contrast, negative parenting behaviors, including control, intrusiveness, negative regard, negative affect, and detachment, predicted lower EF [27]. The PSDQ short version used in this study assessed three parenting styles: authoritative (warmth and support, reasoning, and autonomy granting), authoritarian (physical coercion, verbal hostility, non-reasoning, and punishment), and permissive (indulgence) parenting style. Consistent with previous meta-analytic findings [27], our results reaffirm that components of increased authoritative and reduced authoritarian parenting styles were associated with higher EF. However, our study further highlights the importance of reducing permissive parenting behaviors (e.g., difficulty disciplining the child, spoiling, and giving in to the child’s demands) to better support the development of EF in children. Various parent training programs have been shown to be instrumental in enhancing parents’ self-efficacy, parenting sense of competence, and ameliorating parental mental health problems [51–53]. Importantly, these programs have been associated with reductions in both behavioral and emotional problems in children, ultimately improving parent–child relationships [51, 52]. In light of these findings, it is imperative to consider positive parenting programs as primary interventions in primary pediatric settings from early childhood onward [51]. By addressing parenting practices and supporting parental skills early on, such programs have the potential to not only improve resilience and self-regulatory efficacy, but also mitigate behavioral problems in children during their school years, thus fostering healthier parent–child relationships and promoting overall well-being.

Several limitations must be acknowledged in this study. First, our participants were drawn from a middle to high socioeconomic status within the Thai context, thus limiting the generalizability of our findings to populations of diverse backgrounds. However, similar patterns of relationships have also been observed in children of low socioeconomic backgrounds in a previous study [20], indicating the potential value of our findings in extending understanding across varying contexts with differing levels of early life adversities. Second, the reliance on parent self-reporting to assess parenting behaviors, EF difficulties, and behavioral problems, without observational data, can introduce recall and social desirability biases, as well as a high risk of shared method variance. To mitigate these concerns, self-report data were collected from either parent across multiple time points rather than from a single respondent. Parents were considered reliable informants due to their close and consistent involvement in their child’s development, providing valuable insight into both parenting practices and child behaviors. Therefore, parent-reported data remained essential. The use of data collected across multiple time points, along with moderate to high correlations observed over time, further strengthens the reliability of the findings. Additionally, the incorporation of children’s perspectives through interviews to evaluate resilience and self-regulatory efficacy could have provided unique insights distinct from those of parents. Overall, the longitudinal design of the study, the use of multiple data sources, and the implementation of widely validated questionnaires with different response formats further reduce the likelihood of shared method variance. However, the authors acknowledged potential differences between self-reported parenting measures and observational assessments, as well as possible discrepancies between parent-reported EF-related behaviors and EF performance on cognitive tests. To gain a more nuanced understanding of the relationship between parenting and EF, future studies should consider these factors. Third, the self-regulatory efficacy questionnaire primarily focused on self-regulation within academic tasks and school-related activities, particularly in terms of attention and interference control, executive function, and emotion regulation [24]. Future studies should consider exploring the efficacy of self-regulation in various settings of children’s lives. Fourth, while our findings offered insight into the complex and directional relationships between variables, it is important to note that the causation could not be definitively established due to the longitudinal nature of the study. Fifth, other factors, such as verbal ability, which are typically considered when examining the relationship between parenting and EF, were not measured in this study [54].

Despite these limitations, the strengths were established in its longitudinal cohort design, which facilitated the exploration of complex and long-term associations between EF and parenting behaviors during early childhood, and subsequent outcomes, including resilience, self-regulatory efficacy, and behavioral problems in school-age children. Furthermore, adjustments for several possible confounders, including age, sex, primary caregiver, and maternal education, improved the robustness of our findings.

## Conclusions

In summary, our findings suggested that lower EF difficulties and positive parenting behaviors during preschool were associated with enhanced resilience, improved self-regulatory efficacy, and reduced behavioral problems in school-age children. Interventions aimed at promoting the development of EF, fostering positive parenting practices, and addressing behavioral problems during early childhood have the potential to improve resilience, promote self-regulatory efficacy, and mitigate behavioral problems as children progress through school age.

## Data Availability

The datasets used and/or analyzed during the current study are available from the corresponding author on reasonable request.

## Abbreviations

ABC: Attachment and biobehavioral catch-up intervention
ADHD: Attention-deficit/hyperactivity disorder
BRIEF: Behavior rating inventory of executive function
BRIEF-P: Behavior rating inventory of executive function, preschool version
CBCL: Child behavior checklist
CFI: Comparative fit index
EF: Executive function
GEC: Global executive composite
IOC: Item objective congruence
IQR: Interquartile range
NFI: Normed fit index
PSDQ: Parenting styles and dimensions questionnaire
RCTs: Randomized controlled trials
RMSEA: Root mean square error of approximation
SD: Standard deviation
SDQ: Strengths and difficulties questionnaire

## References

[1] Diamond A. Executive functions. Annu Rev Psychol. 2013;64:135–68. 23020641 10.1146/annurev-psych-113011-143750 PMC4084861

[2] Collins A, Koechlin E. Reasoning, learning, and creativity: frontal lobe function and human decision-making. PLoS Biol. 2012;10: e1001293. 22479152 10.1371/journal.pbio.1001293 PMC3313946

[3] Garon N, Bryson SE, Smith IM. Executive function in preschoolers: a review using an integrative framework. Psychol Bull. 2008;134:31–60. 18193994 10.1037/0033-2909.134.1.31

[4] Hughes C, Ensor R. Executive function and theory of mind: predictive relations from ages 2 to 4. Dev Psychol. 2007;43:1447–59. 18020823 10.1037/0012-1649.43.6.1447

[5] Hughes C, Ensor R. Does executive function matter for preschoolers’ problem behaviors? J Abnorm Child Psychol. 2008;36:1–14. 17914667 10.1007/s10802-007-9107-6

[6] Blair C, Razza RP. Relating effortful control, executive function, and false belief understanding to emerging math and literacy ability in kindergarten. Child Dev. 2007;78:647–63. 17381795 10.1111/j.1467-8624.2007.01019.x

[7] Reilly SE, Downer JT, Grimm KJ. Developmental trajectories of executive functions from preschool to kindergarten. Dev Sci. 2022;25: e13236. 35060244 10.1111/desc.13236 PMC9296695

[8] Blair C, Raver CC, Berry DJ. Family Life Project I Two approaches to estimating the effect of parenting on the development of executive function in early childhood. Dev Psychol. 2014;50:554–65. 23834294 10.1037/a0033647 PMC4682354

[9] Broidy LM, Nagin DS, Tremblay RE, Bates JE, Brame B, Dodge KA, et al. Developmental trajectories of childhood disruptive behaviors and adolescent delinquency: a six-site, cross-national study. Dev Psychol. 2003;39:222–45. 12661883 10.1037//0012-1649.39.2.222 PMC2753823

[10] Yang Y, Shields GS, Zhang Y, Wu H, Chen H, Romer AL. Child executive function and future externalizing and internalizing problems: a meta-analysis of prospective longitudinal studies. Clin Psychol Rev. 2022;97: 102194. 35964337 10.1016/j.cpr.2022.102194

[11] Dodge KA, Lochman JE, Harnish JD, Bates JE, Pettit GS. Reactive and proactive aggression in school children and psychiatrically impaired chronically assaultive youth. J Abnorm Psychol. 1997;106:37–51. 9103716 10.1037//0021-843x.106.1.37

[12] Baler RD, Volkow ND. Drug addiction: the neurobiology of disrupted self-control. Trends Mol Med. 2006;12:559–66. 17070107 10.1016/j.molmed.2006.10.005

[13] Diamond A, Carlson SM, Beck DM. Preschool children’s performance in task switching on the dimensional change card sort task: separating the dimensions aids the ability to switch. Dev Neuropsychol. 2005;28:689–729. 16144433 10.1207/s15326942dn2802_7 PMC1474810

[14] Fairchild G, van Goozen SH, Stollery SJ, Aitken MR, Savage J, Moore SC, et al. Decision making and executive function in male adolescents with early-onset or adolescence-onset conduct disorder and control subjects. Biol Psychiatry. 2009;66:162–8. 19362293 10.1016/j.biopsych.2009.02.024 PMC2733860

[15] Denson TF, Pedersen WC, Friese M, Hahm A, Roberts L. Understanding impulsive aggression: angry rumination and reduced self-control capacity are mechanisms underlying the provocation-aggression relationship. Pers Soc Psychol Bull. 2011;37:850–62. 21421767 10.1177/0146167211401420

[16] Barch DM. The cognitive neuroscience of schizophrenia. Annu Rev Clin Psychol. 2005;1:321–53. 17716091 10.1146/annurev.clinpsy.1.102803.143959

[17] Chmitorz A, Kunzler A, Helmreich I, Tuscher O, Kalisch R, Kubiak T, et al. Intervention studies to foster resilience-a systematic review and proposal for a resilience framework in future intervention studies. Clin Psychol Rev. 2018;59:78–100. 29167029 10.1016/j.cpr.2017.11.002

[18] Sapienza JK, Masten AS. Understanding and promoting resilience in children and youth. Curr Opin Psychiatry. 2011;24:267–73. 21546838 10.1097/YCO.0b013e32834776a8

[19] Masten AS. Global perspectives on resilience in children and youth. Child Dev. 2014;85:6–20. 24341286 10.1111/cdev.12205

[20] Avci G, Hanten G, Schmidt A, Li X, Orsten K, Faber J, et al. Cognitive contributors to resilience in youth from underserved populations: a brief report. J Public Ment Health. 2013;12:165–70.

[21] Taylor ZE, Ruiz Y. executive function, dispositional resilience, and cognitive engagement in Latinx children of migrant farmworkers. Child Youth Serv Rev. 2019;100:57–63.

[22] Bandura A, Barbaranelli C, Caprara GV, Pastorelli C. Self-efficacy beliefs as shapers of children’s aspirations and career trajectories. Child Dev. 2001;72:187–206. 11280478 10.1111/1467-8624.00273

[23] Zimmerman BJ. Self-regulated learning and academic achievement: an overview. Educ Psychol. 1990;25:3–17.

[24] Paananen M, Aro T, Viholainen H, Koponen T, Tolvanen A, Westerholm J, et al. Self-regulatory efficacy and sources of efficacy in elementary school pupils: self-regulatory experiences in a population sample and pupils with attention and executive function difficulties. Learn Individ Differ. 2019;70:53–61.

[25] Bandura A, Barbaranelli C, Caprara GV, Pastorelli C. Multifaceted impact of self-efficacy beliefs on academic functioning. Child Dev. 1996;67:1206–22. 8706518

[26] Tabassam W, Grainger J. Self-concept, attributional style and self-efficacy beliefs of students with learning disabilities with and without attention deficit hyperactivity disorder. Learn Disabil Q. 2002;25:141–51.

[27] Valcan DS, Davis H, Pino-Pasternak D. Parental behaviours predicting early childhood executive functions: a meta-analysis. Educ Psychol Rev. 2018;30:607–49.

[28] Zhang Q, Li JJ. Explaining the prospective association of positive and negative parenting behaviors and child ADHD symptoms: pathways through child executive function and reward responsivity. J Atten Disord. 2022;26:1774–87. 35676827 10.1177/10870547221104079 PMC9960170

[29] Ma Y, Xing X, Zhang M. Parental rejection and school-aged children’s externalizing behavior problems in China: the roles of executive function and callous-unemotional traits. Child Psychiatry Hum Dev. 2024;55:152–63. 35789449 10.1007/s10578-022-01397-6

[30] Valadez EA, Tottenham N, Korom M, Tabachnick AR, Pine DS, Dozier M. A randomized controlled trial of a parenting intervention during infancy alters amygdala-prefrontal circuitry in middle childhood. J Am Acad Child Adolesc Psychiatry. 2024;63:29–38. 37385583 10.1016/j.jaac.2023.06.015 PMC10751390

[31] Korom M, Goldstein A, Tabachnick AR, Palmwood EN, Simons RF, Dozier M. Early parenting intervention accelerates inhibitory control development among CPS-involved children in middle childhood: a randomized clinical trial. Dev Sci. 2021;24: e13054. 33098739 10.1111/desc.13054 PMC8065067

[32] Svendsen S, Griffin J, Forkey H. Using the attachment relationship and positive parenting principles to build childhood resilience. Adv Pediatr. 2020;67:15–28. 32591059 10.1016/j.yapd.2020.04.004

[33] Vijakkhana N, Wilaisakditipakorn T, Ruedeekhajorn K, Pruksananonda C, Chonchaiya W. Evening media exposure reduces night-time sleep. Acta Paediatr. 2015;104:306–12. 25521612 10.1111/apa.12904

[34] Detnakarintra K, Trairatvorakul P, Pruksananonda C, Chonchaiya W. Positive mother-child interactions and parenting styles were associated with lower screen time in early childhood. Acta Paediatr. 2020;109:817–26. 31509278 10.1111/apa.15007

[35] Supanitayanon S, Trairatvorakul P, Chonchaiya W. Screen media exposure in the first 2 years of life and preschool cognitive development: a longitudinal study. Pediatr Res. 2020;88:894–902. 32170192 10.1038/s41390-020-0831-8

[36] Srisinghasongkram P, Trairatvorakul P, Maes M, Chonchaiya W. Effect of early screen media multitasking on behavioural problems in school-age children. Eur Child Adolesc Psychiatry. 2021;30:1281–97. 32856131 10.1007/s00787-020-01623-3

[37] Gioia G, Espy K, Isquith P. Behavior rating inventory of executive function-preschool version (BRIEF-P). Florida: PAR, Inc; 2003.

[38] Gioia GA, Isquith PK, Guy SC, Kenworthy L. Behavior rating inventory of executive function (BRIEF). Florida: PAR, Inc; 2000.

[39] Robinson CC, Mandelco B, Olsen S, Hart C. The parenting styles and dimensions questionnaire (PSDQ) In: Perlmutter BF, Touliatos J, editors. Handbook of family measurement techniques, vol 3 instruments & index. Thousand Oaks: Sage; 2001. p. 319–21.

[40] Achenbach TM, Rescorla LA. Manual for the ASEBA preschool forms & profiles. Burlington: University of Vermont, Research Center for Children, Youth and Families; 2000.

[41] Goodman R, Ford T, Simmons H, Gatward R, Meltzer H. Using the strengths and difficulties questionnaire (SDQ) to screen for child psychiatric disorders in a community sample. Br J Psychiatry. 2000;177:534–9. 11102329 10.1192/bjp.177.6.534

[42] Woerner W, Nuanmanee S, Becker A, Wongpiromsarn Y, Mongkol A. Normative data and psychometric properties of the Thai version of the strengths and difficulties questionnaire (SDQ). J Ment Health Thai. 2011;19:42–57.

[43] Takviriyanun N. Development and testing of the resilience factors scale for Thai adolescents. Nurs Health Sci. 2008;10:203–8. 18786062 10.1111/j.1442-2018.2008.00398.x

[44] Obradović J. Physiological responsivity and executive functioning: implications for adaptation and resilience in early childhood. Child Dev Perspect. 2016;10:65–70.

[45] Dishion TJ, Connell A. Adolescents’ resilience as a self-regulatory process: promising themes for linking intervention with developmental science. Ann NY Acad Sci. 2006;1094:125–38. 17347346 10.1196/annals.1376.012

[46] Kirkorian HL, Pempek TA, Murphy LA, Schmidt ME, Anderson DR. The impact of background television on parent-child interaction. Child Dev. 2009;80:1350–9. 19765004 10.1111/j.1467-8624.2009.01337.x

[47] Nathanson AI, Alade F, Sharp ML, Rasmussen EE, Christy K. The relation between television exposure and executive function among preschoolers. Dev Psychol. 2014;50:1497–506. 24447117 10.1037/a0035714

[48] Linebarger DL, Barr R, Lapierre MA, Piotrowski JT. Associations between parenting, media use, cumulative risk, and children’s executive functioning. J Dev Behav Pediatr. 2014;35:367–77. 25007059 10.1097/DBP.0000000000000069

[49] Radesky JS, Kaciroti N, Weeks HM, Schaller A, Miller AL. Longitudinal associations between use of mobile devices for calming and emotional reactivity and executive functioning in children aged 3 to 5 years. JAMA Pediatr. 2023;177:62–70. 36508199 10.1001/jamapediatrics.2022.4793 PMC9857453

[50] Blair C. Stress and the development of self-regulation in context. Child Dev Perspect. 2010;4:181–8. 21779305 10.1111/j.1750-8606.2010.00145.x PMC3138186

[51] Sanders MR, Kirby JN, Tellegen CL, Day JJ. The Triple P-positive parenting program: a systematic review and meta-analysis of a multi-level system of parenting support. Clin Psychol Rev. 2014;34:337–57. 24842549 10.1016/j.cpr.2014.04.003

[52] Tuntipuchitanon S, Kangwanthiti IO, Jirakran K, Trairatvorakul P, Chonchaiya W. Online positive parenting programme for promoting parenting competencies and skills: randomised controlled trial. Sci Rep. 2024;14:20001. 10.1038/s41598-024-70842-4. 39198492 10.1038/s41598-024-70842-4 PMC11358410

[53] Pornprasitsakul P, Jirakran K, Trairatvorakul P, Chonchaiya W. Parent-focused online video intervention for promoting parenting sense of competence: a randomized controlled trial. Pediatr Res. 2025. 10.1038/s41390-025-03843-2. 39815090 10.1038/s41390-025-03843-2

[54] Hammond SI, Muller U, Carpendale JI, Bibok MB, Liebermann-Finestone DP. The effects of parental scaffolding on preschoolers’ executive function. Dev Psychol. 2012;48:271–81. 21928877 10.1037/a0025519

